# Screening & staging of colorectal cancer

**DOI:** 10.1186/1470-7330-14-S1-O8

**Published:** 2014-10-09

**Authors:** Wolfgang Schima, Anno Graser

**Affiliations:** 1 Department of Diagnostic and Interventional Radiology, Krankenhaus Goettlicher Heiland, KH der Barmherzigen Schwestern, and Sankt Josef-Krankenhaus, Vinzenzgruppe, 1170 Vienna, Austria; 2 Radiologie München, Burgstrasse 7, 80331 München, Germany

## 

In Europe, colorectal cancer (CRC) is the second most common cancer, and the second most common cause of death from cancer [1]. The vast majority of CRC develop from benign precursor lesions, so-called adenomatous polyps, which via the adenoma-carcinoma pathway may eventually transform into colon cancer. It has been shown that endoscopic removal of adenomas interrupts this pathway and subsequently reduces CRC incidence and cancer-related mortality. Thus endoscopic CRC screening programs have been instituted in many countries to reduced CRC cancer mortality. However, limited availability of colonoscopy and limited adherence of the population to colonoscopy-based screening programs is well documented. CT colonography (CTC) has evolved as an effective tool to detect small colorectal polyps, with a high sensitivity to diagnose adenomas ≥ 10 mm and advanced adenomas (with dysplasia) [2]. After negative screening CTC, clinically presenting CRC is rare in the 5 years following CTC [3].

Guidelines for staging of CRC have been developed by the European Society of Medical Oncology (ESMO) [4], which recommend MRI and/or endorectal ultrasound for local staging of rectal cancer (in order to decide which patients need neoadjuvant therapy). MRI is preferred in stenotic tumors or cancers in the upper third of the rectum. In patients with colon cancer, local staging (by CT) primarily seeks to exclude T4 disease with infiltration into other organs. Variability exists in different European countries regarding the use of contrast-enhanced MDCT of the abdomen and chest (to be preferred over chest X-ray) for evaluation of nodal disease and distant metastases. Assessment of lymph nodes based on size criteria alone has some limitations, because metastases can be found even in normal-sized lymph nodes [5,6]. FDG-PET is not recommended for staging [4]. It might be used for staging of patients with CT-detected synchronous liver metastases scheduled for liver surgery. However, a recent study showed that PET/CT in patients with potentially resectable liver metastases did not result in frequent change of management and did not improve overall survival [7]. CTC screening for CRC will be presented and CRC staging by MDCT and MRI will be highlighted.
